# Daratumumab in Transplant-Ineligible Newly Diagnosed Multiple Myeloma: A Meta-Analysis of Randomized Controlled Trials

**DOI:** 10.3390/cancers17203349

**Published:** 2025-10-17

**Authors:** Chi Wang, Zhengyang Xu, Meilin Jiang, Yuzhe Chen, Yu Lan

**Affiliations:** State Key Laboratory of Experimental Hematology, Key Laboratory for Regenerative Medicine of Ministry of Education, Institute of Hematology, School of Medicine, Jinan University, Guangzhou 510632, China

**Keywords:** daratumumab, multiple myeloma, transplant-ineligible, progression-free survival, adverse events, meta-analysis

## Abstract

Background: Treatment for transplant-ineligible (TIE) newly diagnosed multiple myeloma (NDMM) has improved with anti-CD38 monoclonal antibodies. Among them, daratumumab, combined with standard therapies, has shown promising results in clinical trials. This meta-analysis consolidates evidence on the effectiveness and safety of daratumumab-based treatments for this patient group from all available randomized controlled trials (RCTs). Methods: We systematically searched PubMed, Embase, Cochrane Central Register of Controlled Trials, and clinical trial registries from inception to September 2025 for phase II and III RCTs comparing daratumumab-containing regimens to non-daratumumab controls in TIE NDMM patients. Primary outcomes were progression-free survival (PFS) and overall survival (OS). Secondary outcomes included minimal residual disease (MRD) negativity rate and adverse events (AEs). Heterogeneity was assessed using I^2^ statistics, and subgroup analyses were performed to explore potential sources of heterogeneity. Results: Six RCTs involving 2478 patients were included. Daratumumab-based regimens significantly improved PFS (hazard ratio [HR] = 0.544, 95% confidence interval [CI]: 0.483–0.612, *p* < 0.001; I^2^ = 28.6%) and OS (HR = 0.693, 95% CI: 0.606–0.791, *p* < 0.001; I^2^ = 30.6%). The MRD negativity rate was significantly higher with daratumumab (risk ratio [RR] = 2.322, 95% CI: 1.486–3.627, *p* < 0.001). Furthermore, daratumumab-based regimens yielded a four-fold increase in the rate of sustained MRD negativity (≥12 months) (RR = 3.999, 95% CI: 1.094–8.403, *p* < 0.001). However, these regimens were associated with increased risks of serious adverse events (SAEs) (RR = 1.146, 95% CI: 1.064–1.233, *p* < 0.001), overall grade 3/4 AEs (RR = 1.075, 95% CI: 1.038–1.115, *p* < 0.001), neutropenia, lymphopenia, infections, pneumonia, and fatal AEs. No significant differences were observed in thrombocytopenia or anemia. Conclusions: Daratumumab-based regimens significantly improve survival outcomes and the depth/durability of treatment response in TIE NDMM patients, supporting their use as first-line therapy. However, the increased risk of specific AEs necessitates careful patient selection, proactive infection prevention, and vigilant monitoring. These findings provide robust evidence for clinical practice guidelines and underscore the need to balance efficacy with safety in this vulnerable population.

## Simple Summary

Multiple myeloma is a type of blood cancer that often affects older adults, many of whom are not eligible for an intensive stem cell transplant. For these patients, finding treatments that are both effective and tolerable is crucial. Daratumumab is a targeted drug that has been added to standard therapies to improve outcomes. By combining data from all relevant randomized controlled trials, this study confirms that treatment plans containing daratumumab significantly delay cancer progression, help patients live longer, and increase the chances of achieving deep remission where no cancer cells can be detected. However, these powerful regimens also come with an increased risk of serious side effects, particularly infections. Therefore, while daratumumab-based therapy is a highly effective first-line option for transplant-ineligible patients, its use requires careful management and monitoring to maximize benefits and minimize risks.

## 1. Introduction

Multiple myeloma (MM) is a significant hematologic cancer characterized by malignant plasma cells in the bone marrow. It accounts for 1–2% of all cancers and 10–15% of hematologic malignancies, with a median diagnosis age of 70 years [1,2]. This demographic reality means that a substantial proportion of patients present with age-related frailty, comorbidities, and reduced functional reserve, rendering them unsuitable for the intensive and myelosuppressive conditioning regimens required for autologous stem cell transplantation (ASCT) [3]. Treating transplant-ineligible (TIE) newly diagnosed multiple myeloma (NDMM) patients requires a careful balance of treatment effectiveness, tolerability, and the goal of sustained remission. The therapeutic goal in this population has thus shifted from palliation to achieving durable, deep remissions that maximize both the duration and quality of life.

Over the past two decades, treatment options for TIE NDMM have evolved significantly. The introduction of proteasome inhibitors (e.g., bortezomib) and immunomodulatory drugs (e.g., lenalidomide), used with dexamethasone, moved care beyond traditional chemotherapy [4,5]. Modern standard regimens—such as lenalidomide plus dexamethasone (Rd), bortezomib plus melphalan and prednisone (VMP), and bortezomib plus lenalidomide and dexamethasone (VRd)—have improved progression-free survival (PFS) and overall survival (OS) [6,7]. Despite these advances, disease progression remains inevitable, and the long-term outcomes for many patients remain suboptimal, prompting the search for more effective treatments.

A major subsequent advancement has been the development of monoclonal antibodies targeting CD38, a protein highly expressed on myeloma cells [8]. Daratumumab, a first-in-class anti-CD38 antibody, kills cancer cells through multiple immune-mediated mechanisms, including antibody-dependent cellular cytotoxicity (ADCC), complement-dependent cytotoxicity (CDC), antibody-dependent cellular phagocytosis (ADCP), and the induction of apoptosis. It has transformed MM management for both newly diagnosed and relapsed patients [9,10,11]. It is noteworthy that isatuximab, another anti-CD38 monoclonal antibody, has also demonstrated significant efficacy in TIE NDMM, as evidenced by the IMROZ trial [12].

Several pivotal phase III randomized controlled trials (RCTs) have established the efficacy of daratumumab-based regimens for TIE patients. For instance, the MAIA trial showed the superiority of daratumumab with lenalidomide and dexamethasone (DRd) over Rd alone [13,14,15], and the ALCYONE trial demonstrated benefits for daratumumab with VMP (D-VMP) over VMP [16,17]. These findings have led global guidelines to recommend daratumumab combinations as preferred frontline options.

While individual trials consistently show superior efficacy, each is powered primarily for its primary endpoint, often PFS. The consistency of the overall survival benefit and the full safety profile across a broader patient population requires further synthesis. Individual trials may also be underpowered to detect significant differences in less common adverse events (AEs). Although previous meta-analyses have explored anti-CD38 antibodies, including a recent Cochrane systematic review, none have provided a comprehensive, updated analysis focused exclusively on all RCTs for daratumumab in frontline TIE NDMM that integrates mature efficacy data with a detailed safety assessment [18,19,20]. The Cochrane review included four trials (ALCYONE, MAIA, OCTANS, and AMaRC 03-16) and demonstrated significant benefits in OS and PFS but was limited by the number of studies and lacked data from recent trials such as CEPHEUS and GEM-2017FIT. Moreover, a granular analysis of sustained MRD negativity—a key prognostic marker for long-term outcomes—and a detailed, quantitative synthesis of serious and fatal AEs across the entire evidence base are lacking.

To address this gap, we conducted this systematic meta-analysis. Our objective is to synthesize the efficacy data (including PFS, OS, and minimal residual disease (MRD) negativity rates) from all published RCTs evaluating daratumumab-based therapies in TIE NDMM patients. Crucially, we also provide a detailed quantification of associated risks, including serious AEs, to offer a comprehensive benefit–risk assessment for optimizing treatment in this vulnerable population. By incorporating the most recent trial data and performing robust subgroup and sensitivity analyses, this study aims to provide the most current and definitive evidence to guide clinical decision-making.

## 2. Materials and Methods

### 2.1. Search Strategy and Study Selection

This meta-analysis was conducted in accordance with Preferred Reporting Items for Systematic Reviews and Meta-Analyses (PRISMA) guidelines (Table S1) and has not been registered [21]. A systematic literature search was performed across PubMed, Embase, Cochrane Central Register of Controlled Trials, and clinical trial registries (ClinicalTrials.gov) from inception to September 2025. To identify relevant studies, the search protocol entailed using both medical subject headings (MeSH) and free-text terms related to “multiple myeloma,” “daratumumab,” “newly diagnosed,” and “transplant-ineligible.” No language restrictions were applied. Studies were included if they met the following criteria: (1) phase II or III RCTs; (2) participants with TIE NDMM; (3) intervention groups receiving daratumumab-containing regimens; (4) control groups receiving standard therapies without daratumumab; and (5) reporting of at least one outcome of interest. Exclusion criteria included: (1) non-randomized studies; (2) studies including transplant-eligible patients; (3) duplicate publications; and (4) studies with insufficient data for meta-analysis.

### 2.2. Data Extraction and Quality Assessment

Two investigators independently extracted data using a standardized form, resolving disagreements by consensus or with a third reviewer. Extracted information included study metadata, participant characteristics, intervention details, and outcome data (PFS, OS, MRD negativity, and AEs). For safety outcomes, data on treatment-emergent adverse events (TEAEs) were extracted from the most detailed available report for each trial, which for key AEs was consistent with the longest follow-up. Specifically, serious adverse events (SAEs) were analyzed based on the definition provided by each individual trial, which typically encompasses events resulting in death, are life-threatening, require inpatient hospitalization or prolongation of existing hospitalization, result in persistent or significant disability/incapacity, or are important medical events. Study quality was assessed using the Cochrane Risk of Bias tool for RCTs [22].

### 2.3. Statistical Analysis

For time-to-event outcomes, such as PFS and OS, hazard ratios (HRs) and their corresponding 95% confidence intervals (CIs) were pooled directly from the published results of each study. For dichotomous outcomes (MRD negativity and AEs), risk ratios (RRs) with 95% CIs were calculated.

Pooled estimates were calculated using a DerSimonian-Laird random-effects model or a fixed-effect model (Mantel–Haenszel method), depending on the presence or absence of heterogeneity. The degree of heterogeneity among the studies was quantified by applying the I^2^ statistic. Conventional interpretation thresholds were employed, where I^2^ values of 25%, 50%, and 75% indicated low, moderate, and substantial heterogeneity, respectively. To explore potential sources of variation, subgroup analyses were carried out according to key disease characteristics (ISS stage [I, II, III], cytogenetic risk [standard vs. high], Eastern Cooperative Oncology Group [ECOG] performance status [0 vs. 1 or 2], and renal function [creatinine clearance ≤ 60 mL/min vs. >60 mL/min]).

The stability of the pooled results was evaluated through sensitivity analyses, which involved successively omitting individual studies. Although the use of funnel plots and Egger’s regression test had been pre-specified to evaluate publication bias, these methods were ultimately not utilized. This decision was based on the limited number of studies (n = 6) included in the final meta-analysis, as the statistical power of these tests is known to be insufficient for meaningful interpretation in such small samples [23]. Consequently, publication bias was not formally assessed. All statistical analyses were performed using Stata (version 14.2) (StataCorp, College Station, TX, USA) and RevMan (version 5.4) (The Cochrane Collaboration, Oxford, UK). A two-sided *p*-value < 0.05 was considered statistically significant for all analyses except heterogeneity testing, where *p* < 0.10 was considered significant.

## 3. Results

### 3.1. Study Selection and Characteristics

**Figure 1:**
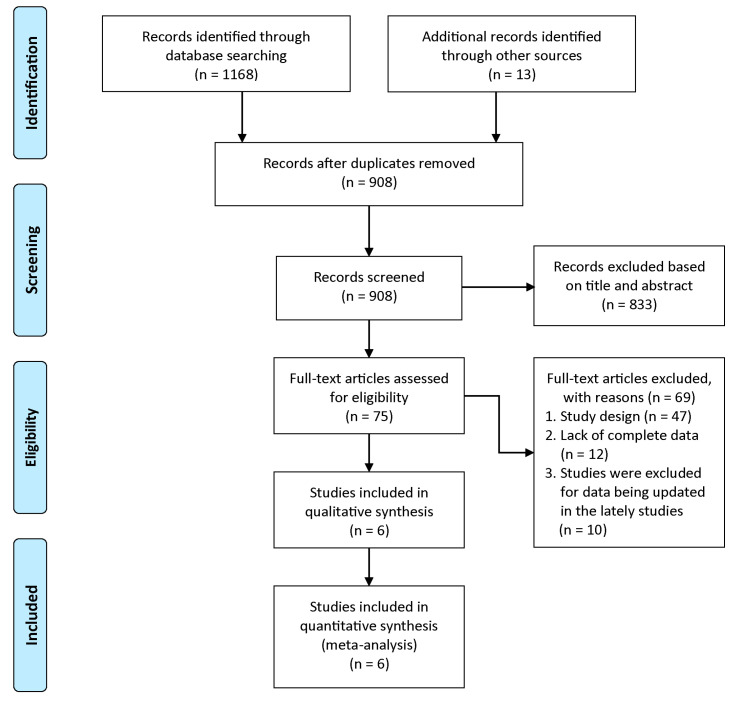
Flow chart of PRISMA.

The systematic literature search identified 1181 potentially relevant records. After removing duplicates and screening titles and abstracts, 75 full-text articles were assessed for eligibility. Six RCTs involving 2478 patients met the inclusion criteria. The study selection process is detailed in a PRISMA flow diagram (Figure 1). Daratumumab was combined with various standard regimens: D-VMP in ALCYONE and OCTANS [16,17,24,25]; D-Rd in MAIA [14,15]; bortezomib–cyclophosphamide–dexamethasone (D-VCD) in AMaRC 03-16 [26]; carfilzomib–lenalidomide–dexamethasone (D-KRd) in GEM-2017FIT [27]; and bortezomib–lenalidomide–dexamethasone (D-VRd) in CEPHEUS [28]. Control regimens matched the backbone therapies without daratumumab. Patient characteristics were generally balanced across studies. The median age ranged from 69 to 76 years, reflecting the typical age of the TIE population. Notable differences in inclusion criteria were observed, particularly regarding renal function; for example, the ALCYONE trial required a creatinine clearance (CrCl) of ≥40 mL/min, while the MAIA and AMaRC 03-16 trials allowed inclusion of patients with a CrCl of ≥30 mL/min, reflecting varied approaches in clinical practice towards patients with renal impairment (Table 1).

### 3.2. Efficacy Outcomes

**Figure 2:**
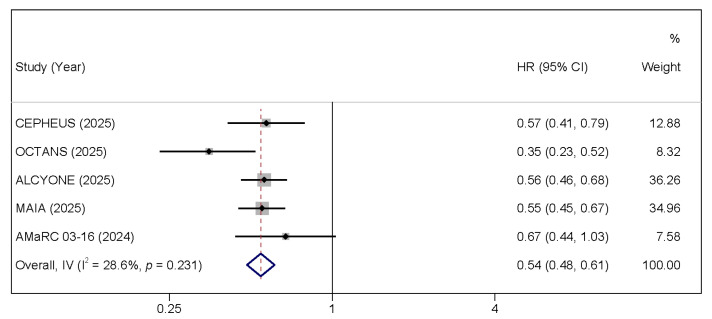
Forest plots of HRs for PFS comparing daratumumab-based regimens to the control arm [15,16,24,26,27,28].

**Figure 3:**
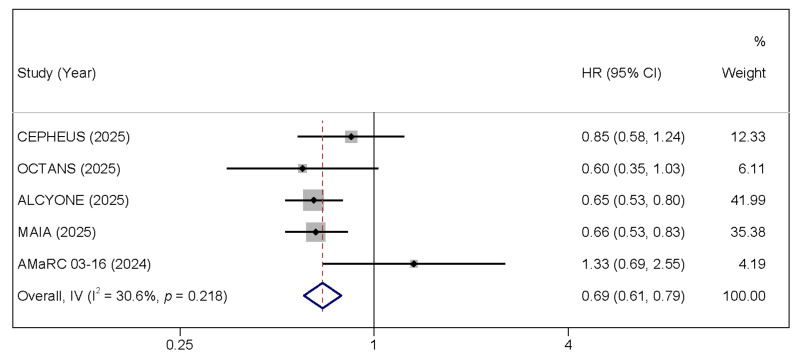
Forest plots of HRs for OS comparing daratumumab-based regimens to the control arm [15,16,24,26,27,28].

All six included studies reported PFS. As patients in the GEM-2017FIT trial were randomized (1:1:1) to VMP, KRd, or D-KRd, no direct comparison was made between KRd and D-KRd; therefore, PFS analysis was performed using data from five trials [27]. Daratumumab-based regimens significantly reduced the risk of disease progression or death compared with control therapies (pooled HR = 0.544, 95% CI: 0.483–0.612, *p* < 0.001), with moderate heterogeneity observed across studies (I^2^ = 28.6%, *p* = 0.231; Figure 2). This translates to a 45.6% reduction in the risk of progression or death, a substantial and clinically meaningful improvement. Similarly, OS data were available from five trials. Pooled analysis demonstrated a significant OS benefit favoring daratumumab-containing regimens (HR = 0.693, 95% CI: 0.606–0.791, *p* < 0.001), with moderate heterogeneity (I^2^ = 30.6%, *p* = 0.218; Figure 3). This represents a 30.7% reduction in the risk of death, confirming that the PFS benefit translates into a significant overall survival advantage for TIE NDMM patients.

**Figure 4:**
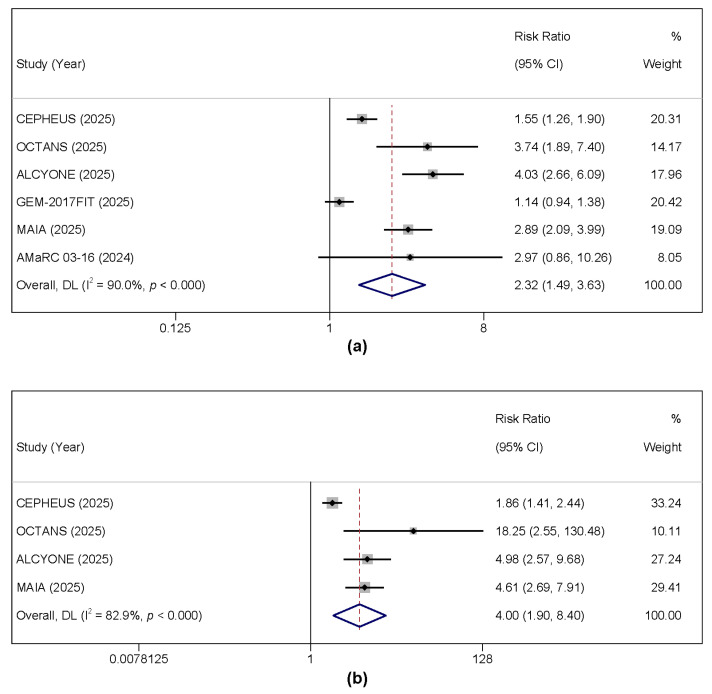
Forest plots of RRs for MRD response comparing daratumumab-based regimens to the control arm. (**a**) MRD negativity rate. (**b**) Sustained MRD negativity (≥12 months) [15,16,24,26,27,28].

MRD negativity rates at a sensitivity threshold of 10^5^ were reported in all included studies. A total of 500 out of 1278 (39.1%) patients in the daratumumab group achieved MRD negativity, compared with 238 out of 1208 (19.7%) in the control group. Daratumumab-based therapy was associated with a significantly higher MRD negativity rate (RR = 2.322, 95% CI: 1.486–3.627, *p* < 0.001). Substantial heterogeneity was detected (I^2^ = 90%, *p* < 0.001); thus, a random-effects model was used for this analysis (Figure 4a). Sustained MRD negativity (defined as MRD-negative status maintained for ≥12 months) is a key predictor of long-term outcomes. This was a pre-specified or exploratory endpoint in four of the six trials (MAIA, ALCYONE, CEPHEUS, OCTANS). We performed a meta-analysis on this endpoint, which demonstrated that daratumumab-based regimens significantly increased the rate of sustained MRD negativity by approximately four-fold compared to control therapies (pooled RR = 3.999, 95% CI: 1.094–8.403, *p* < 0.001; Figure 4b). Substantial heterogeneity was observed (I^2^ = 82.9%, *p* = 0.001), likely reflecting differences in study populations, backbone regimens, and follow-up duration. Despite this heterogeneity, the magnitude and consistency of the effect underscore the ability of daratumumab to not only deepen responses but also to confer remarkable durability (Figure 4b). Achieving sustained MRD negativity is increasingly recognized as a surrogate for long-term PFS and OS, making this finding particularly significant.

### 3.3. Safety Outcomes

Treatment with daratumumab was associated with a higher risk of SAEs (RR = 1.146, 95% CI: 1.064–1.233, *p* < 0.001), overall grade 3/4 AEs; RR = 1.075, 95% CI: 1.038–1.115, *p* < 0.001 and fatal AEs, defined as on-study deaths from any cause (RR = 1.439, 95% CI: 1.104–1.876, *p* = 0.007). The significant increase in SAEs, which captures a broader range of clinically significant toxicities beyond grade 3/4 laboratory abnormalities, underscores the substantial treatment burden associated with daratumumab-containing regimens. While the point estimate for overall grade 3/4 AEs indicates a modest increase in risk, the risk of fatal AEs was more substantial. It is important to note that this analysis for fatal AEs included all on-study mortality, encompassing deaths from disease progression and comorbidities in addition to potential treatment-related fatalities. Consequently, a formal analysis specifically assessing treatment-related deaths was not possible due to inconsistent causality reporting across the included trials. Significantly increased risks were also observed for grade 3/4 lymphopenia (RR = 1.285, 95% CI: 1.028–1.605, *p* = 0.028), infections (RR = 1.429, 95% CI: 1.144–1.784, *p* = 0.002), and pneumonia (RR = 1.760, 95% CI: 1.102–2.812, *p* = 0.018). A trend toward increased neutropenia was observed (RR = 1.253, 95% CI: 0.987–1.590, *p* = 0.065). No significant differences were observed in the incidence of thrombocytopenia (RR = 0.951, *p* = 0.476) and anemia (RR = 0.937, *p* = 0.476) between the two groups (Table 2).

### 3.4. Subgroup and Sensitivity Analyses

Subgroup analyses were conducted based on disease characteristics, including ISS stage, cytogenetic risk, ECOG performance status, and CrCl. Due to the limited number of studies available for each subgroup, only PFS was analyzed. The PFS benefit associated with daratumumab was consistently observed across all subgroups (Figures S1–S4).

Leave-one-out sensitivity analysis was performed for all outcomes. The efficacy outcomes, including PFS, OS, MRD negativity rate, sustained MRD negative rate, and SAEs, remained robust and statistically significant upon sequential removal of each individual study, confirming the stability of these results. In contrast, certain safety outcomes were sensitive to the exclusion of specific trials. Upon removal of the MAIA trial, the increased risk of overall grade 3/4 AEs (RR = 1.062, 95% CI: 0.988−1.142, *p* = 0.104) and grade 3/4 lymphopenia (RR = 1.188, 95% CI: 0.898−1.571, *p* = 0.227) were no longer statistically significant. Furthermore, when the OCTANS trial was excluded, the risk of grade 3/4 neutropenia became statistically significant (RR = 1.253, 95% CI: 1.012−1.552, *p* = 0.039; Table S2). These findings indicate that the pooled efficacy estimates are highly robust, while some safety outcomes may be influenced by the results of individual studies. This highlights the importance of considering the entire body of evidence when evaluating the safety profile.

### 3.5. Risk of Bias Assessment

**Figure 5:**
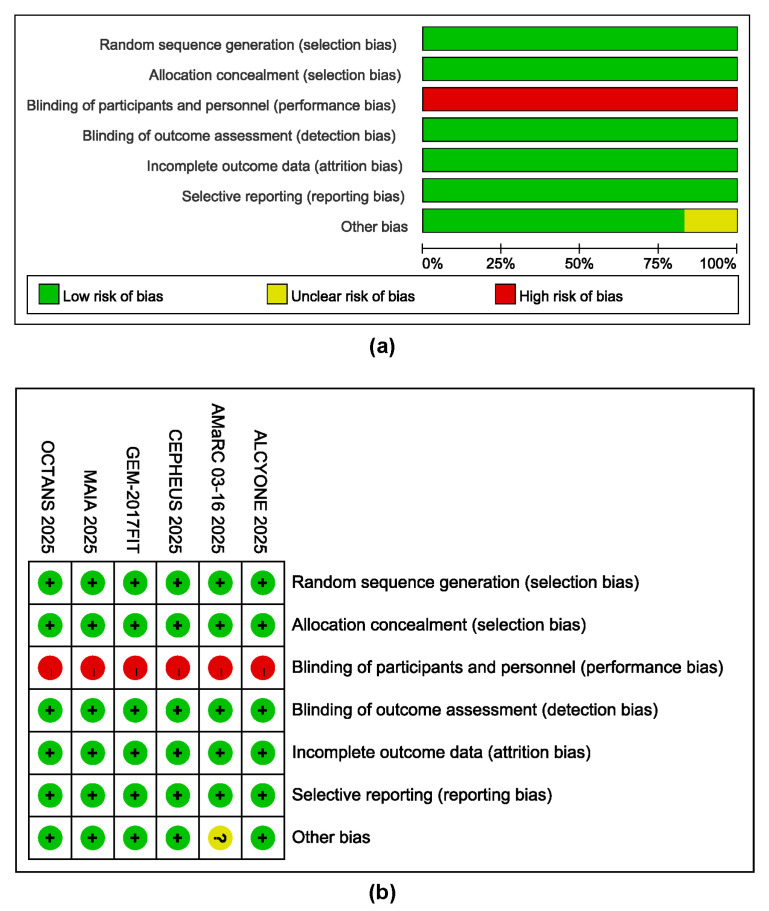
Appraisal of the bias in the included studies. (**a**) Total bias risk in all studies. (**b**) Summary of bias in the individual study [15,16,24,26,27,28].

All included studies were open-label designs due to the nature of daratumumab administration. Therefore, blindness was considered the main risk of bias in our study. The randomization process was adequately described in all trials, with low risk of bias. Performance bias was deemed high risk across all studies due to the open-label design. Detection bias raised some concerns for subjective outcomes but posed a low risk for objective outcomes, such as survival. Attrition bias was low risk in most studies, with complete follow-up data. Reporting bias was low risk, as all pre-specified outcomes were reported. Overall, the risk of bias was defined as moderate to low (Figure 5).

## 4. Discussion

This comprehensive meta-analysis of six RCTs demonstrates that daratumumab-based regimens significantly improve survival outcomes and depth of response in TIE NDMM. The consistent benefits across different treatment combinations and patient subgroups reinforce the value of daratumumab in first-line treatment.

Our findings are consistent with the recent Cochrane review, which also reported significant improvements in OS (HR 0.64) and PFS (HR 0.48) with daratumumab-based regimens [20]. The present analysis extends these findings by incorporating two additional trials (CEPHEUS and GEM-2017FIT) and providing longer follow-up data, thereby strengthening the overall evidence base. Furthermore, we provide a more granular safety profile, confirming the increased risks of infections, pneumonia, and lymphopenia associated with daratumumab, which aligns with the Cochrane review’s conclusion regarding serious AEs. Pooled results from our meta-analysis showed a 45.6% reduction in the risk of disease progression or death (HR = 0.544) and a 30.7% reduction in the risk of mortality (HR = 0.693) with daratumumab-containing therapies, corroborating the robust treatment effect observed in previous meta-analyses. Additionally, daratumumab-based regimens were associated with a significantly higher rate of MRD negativity (RR = 2.322; 39.1% vs. 19.7% in controls), reinforcing its capacity to induce deep and durable responses—a well-established surrogate for long-term survival in multiple myeloma.

The safety profile identified in this analysis is consistent with the known toxicity spectrum of daratumumab [29], which is mechanistically linked to its depletion of CD38-positive immune cells, leading to immunosuppression [30]. We observed increased risks of SAEs, grade 3/4 hematologic toxicities, particularly lymphopenia and infections (including pneumonia), as well as a higher incidence of fatal AEs. The increased risk of SAEs provides a comprehensive view of the treatment’s impact on patient health, reinforcing that the efficacy benefits come with a quantifiable increase in clinically significant morbidity. The increased risk of fatal AEs, which includes deaths from any cause, is a serious finding that warrants careful consideration and must be weighed against the significant survival benefit. The increased risk of infections and fatal events is a serious finding that warrants careful consideration and must be weighed against the significant survival benefit. These results highlight the necessity of implementing proactive preventive strategies and exercising particular caution in frail patients.

An important consideration when evaluating treatment regimens with an increased risk of adverse events is their impact on patient-reported quality of life (QoL). Beyond the incidence of AEs, understanding the net effect on a patient’s daily functioning and well-being is crucial for a comprehensive benefit–risk assessment. While a formal meta-analysis of QoL data was precluded by heterogeneous reporting across trials, available data provide valuable insights. In the CEPHEUS trial, EORTC QLQ-C30 assessments indicated that global health status improved over time in both the D-VRd and VRd groups, with no detrimental effect observed from the addition of daratumumab [28].

The most robust QoL evidence comes from the final analysis of the ALCYONE trial. A mixed-effects model analysis demonstrated that treatment with D-VMP led to statistically significant and clinically meaningful improvements in key patient-reported outcomes compared to VMP alone. Patients receiving D-VMP reported significantly greater improvements from baseline in global health status (LS mean difference vs. VMP: +3.5 points at Month 3, *p* < 0.05; +6.5 points at Month 9, *p* < 0.05), a greater reduction in pain (LS mean difference vs. VMP: −3.3 points at Month 3; −5.6 points at Month 9, *p* < 0.05), and a trend towards better physical functioning. These benefits were even more pronounced in the subgroup of patients with bone lesions at baseline, who experienced significantly greater improvements in global health status (LS mean difference: +3.4 points at Month 3, *p* < 0.05) and a substantially larger reduction in pain (LS mean difference: −4.4 points at Month 3) with D-VMP.

These findings collectively suggest that the profound efficacy benefits and superior disease control afforded by daratumumab-based regimens, including delayed progression and deep responses, effectively translate into preserved or improved QoL. This positive impact on how patients feel and function provides essential context for the observed toxicity profile and supports the overall clinical value of these treatments. Proactive management of AEs, particularly infections, remains vital to maintaining this beneficial balance. Future studies should incorporate standardized and comprehensive QoL assessments to further define the net patient benefit of modern anti-myeloma therapies.

The mechanisms underlying daratumumab’s efficacy in TIE NDMM patients are multifaceted. Beyond its direct anti-myeloma effects through antibody-dependent cellular cytotoxicity, complement-dependent cytotoxicity, and antibody-dependent cellular phagocytosis, daratumumab also alters the immune microenvironment. This is achieved through the depletion of immunosuppressive cell populations and a subsequent enhancement of T-cell activity [9,31,32]. These immunomodulatory effects may be particularly beneficial in elderly patients who often exhibit immune senescence. Furthermore, the synergy between daratumumab and immunomodulatory drugs like lenalidomide is well-documented, as lenalidomide can further enhance immune effector cell function, creating a potent anti-myeloma immune response.

Several clinical implications arise from our findings. Daratumumab should be regarded as a standard component of first-line therapy for TIE NDMM patients, with the choice of combination regimen individualized based on patient fitness, comorbidities, and local availability. For fitter patients, more intensive regimens like D-VRd or D-KRd may be appropriate to maximize depth of response, while for frailer patients, the well-tolerated all-oral D-Rd regimen represents an excellent option. Vigilant monitoring and proactive management of infectious complications are essential. This includes patient education, vaccination (where appropriate), and a low threshold for investigating febrile episodes. The sustained efficacy over extended follow-up supports continuing daratumumab maintenance until disease progression in suitable patients.

This study has several limitations. First, the inclusion of open-label trials may introduce potential bias. Second, variability in follow-up duration across studies could affect the assessment of long-term outcomes. Third, the limited number of studies precludes definitive conclusions regarding specific regimen subtypes or a formal evaluation of publication bias. Fourth, the lack of individual patient data restricted deeper exploration of effect modifiers within specific subgroups.

Furthermore, this meta-analysis was intentionally restricted to daratumumab-based regimens to ensure intervention homogeneity, which necessarily excluded other anti-CD38 antibodies such as isatuximab. While the pivotal IMROZ trial established the efficacy of isatuximab-based therapy (Isa-VRd) in this population [12], and its findings align with the class effect of anti-CD38 antibodies, its inclusion was beyond the scope of our focused analysis. Future comparative studies will be valuable to delineate differences between these agents. Finally, a key limitation of our MRD analysis stems from the varying assessment timepoints across trials, which complicates direct comparison. Despite this heterogeneity, the finding that daratumumab more than doubles the rate of MRD negativity remains robust. Moreover, our analysis of sustained MRD negativity—a more stringent endpoint—provides compelling evidence that daratumumab quadruples the likelihood of achieving a deep, durable remission, reinforcing its capacity to alter the disease course in TIE NDMM patients.

Future research should prioritize identifying predictive biomarkers of response to daratumumab, optimizing combination strategies to maximize efficacy while minimizing toxicity, and developing effective management strategies for daratumumab-related AEs. Studies exploring dose adjustments or alternative schedules for frail patients are urgently needed. Real-world studies will be valuable to generalize these findings to broader clinical populations and diverse healthcare settings. Additionally, cost-effectiveness analyses will be crucial to inform healthcare policy and ensure equitable access to these highly effective but costly therapies.

## 5. Conclusions

This meta-analysis provides robust evidence that daratumumab-based regimens significantly improve PFS, OS, and MRD negativity rate in TIE NDMM patients. The profound and durable responses, including a four-fold increase in sustained MRD negativity, establish daratumumab as a transformative agent in the frontline setting. Although associated with an increased risk of specific AEs, including infections and a modest increase in fatal events, the overall benefit–risk profile supports the use of daratumumab as a cornerstone of first-line therapy in this population. These results reinforce current guideline recommendations. Successful implementation in clinical practice requires an integrated approach that combines these potent regimens with rigorous supportive care, proactive infection prevention, and careful patient selection to maximize survival gains while mitigating treatment-related risks.

## Figures and Tables

**Table 1:** The baseline characteristics of studies included in the meta-analysis.

Study	Year	Phase	Patients		Median Age (Year)		Intervention		Median Follow-Up Time (Months)	Primary End-Point
			E	C	E	C	E	C		
CEPHEUS [28]	2025	III	197	198	70 (42–79)	70 (31–80)	D-VRd	VRd	58.7	MRD
OCTANS [24,25]	2025	III	146	74	69 (58–81)	69 (57–84)	D-VMP	VMP	41.2	≥VGPR
ALCYONE [16,17]	2025	III	350	356	71 (40–93)	71 (50–91)	D-VMP	VMP	86.7	PFS
* GEM-2017FIT [27]	2025	III	153	154	73 (71–76)	73 (71–76)	D-KRd	KRd	33.15	MRD
MAIA [14,15]	2025	III	368	369	73 (70–78)	74 (70–78)	D-Rd	Rd	64.5	PFS
AMaRC 03-16 [26]	2024	II	64	57	75.9 (64–91)	75.4 (62–89)	D-VCD	VCD	44.7	PFS

E: Experimental arm; C: Control arm. * In the GEM-2017FIT trial, patients were randomized 1:1:1 to VMP, KRd, or D-KRd. For this analysis, only the D-KRd vs. KRd comparison was included. D: Daratumumab; V: Bortezomib; R: Lenalidomide; d: Dexamethasone; M: Melphalan; P: Prednisone; K: Carfilzomib; C: Cyclophosphamide.

**Table 2:** Safety profile of the group with daratumumab-based treatment regimens compared to the control group in NDMM patients.

Outcomes	Included Studies ^a^ [14,15,16,24,25,26,27,28]	Pooled Effect Sizes			Heterogeneity	
		RR	95% CI	*p-*Value	I^2^	*p*-Value of Q Test
Fatal AEs	COAlGMAm	1.439	1.104–1.876	0.007	16.0%	0.311
SAEs	COAlMAm	1.146	1.064–1.233	<0.001	0.0%	0.504
Grade 3/4 AEs	OAlMAm	1.075	1.038–1.115	<0.001	0.0%	0.802
Grade 3/4 Lymphopenia	COAlM	1.285	1.028–1.605	0.028	0.0%	0.8
Grade 3/4 Neutropenia	COAlGMAm	1.253	0.987–1.59	0.065	80.5%	<0.001
Grade 3/4 Thrombocytopenia	COAlGMAm	0.951	0.83–1.091	0.476	0.0%	0.978
Grade 3/4 Anemia	COAlGMAm	0.937	0.784–1.12	0.476	33.0%	0.201
Grade 3/4 Infection	COAlGM	1.429	1.144–1.784	0.002	62.3%	0.031
Grade 3/4 Pneumonia	COAlM	1.76	1.102–2.812	0.018	74.9%	0.007

^a^ C: study of CEPHEUS; O: study of OCTANS; Al: study of ALCYONE; G: study of GEM-2017FIT; M: study of MAIA; Am: study of AMaRC 03-16.

## Supplementary Materials

The following supporting information can be downloaded at: https://www.mdpi.com/article/10.3390/cancers17203349/s1. Figure S1: Subgroup analysis of PFS based on IGG stage; Figure S2: Subgroup analysis of PFS based on cytogenetic risk; Figure S3: Subgroup analysis of PFS based on cytogenetic ECOG performance status; Figure S4: Subgroup analysis of PFS based on cytogenetic creatinine clearance; Table S1: PRISMA 2020 Checklist; Table S2: Leave-one-out sensitivity analysis of outcomes.
