# Supplementary material for: Daratumumab in Transplant-Ineligible Newly Diagnosed Multiple Myeloma: A Meta-Analysis of Randomized Controlled Trials
Source: Cancers (Basel). 2025 Oct 17;17(20):3349. doi: 10.3390/cancers17203349 (PMC12563183; doi:10.3390/cancers17203349)
Supplement: Supplementary file 1 [file cancers-17-03349-s001.zip › cancers-3912367-supplementary.pdf]

Supplementary Materials

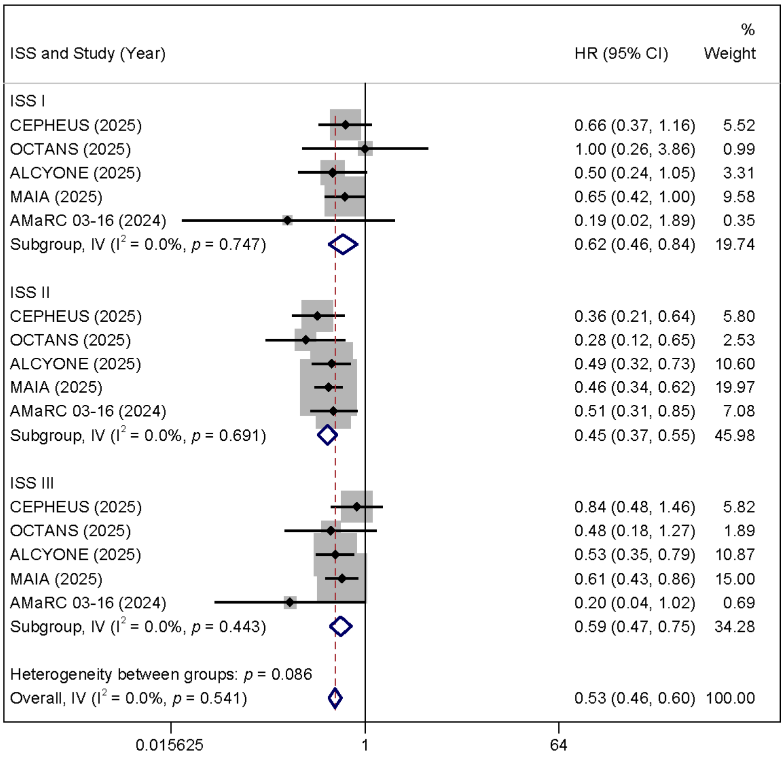

Figure S1. Subgroup analysis of PFS based on IGG stage [15, 16, 24, 26].

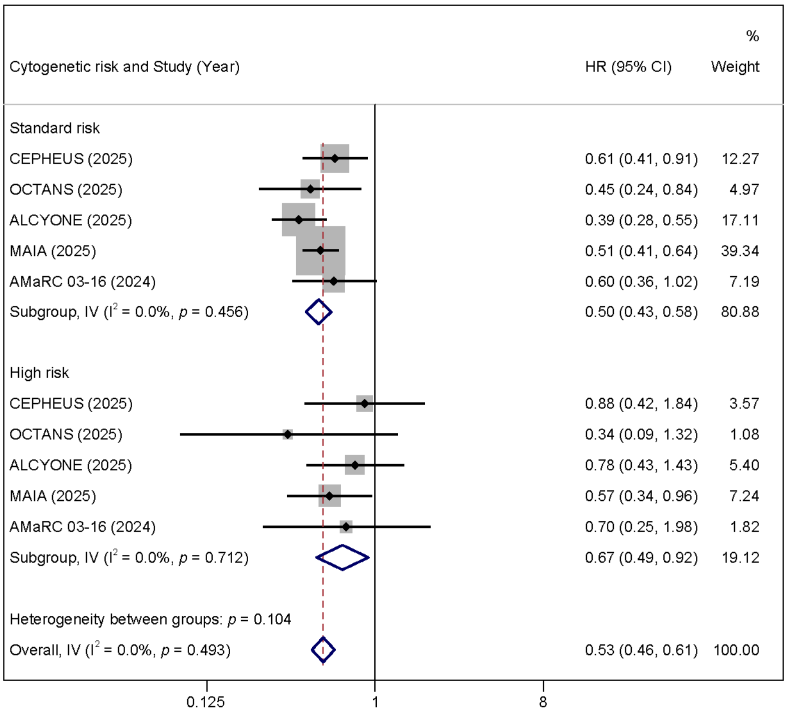

Figure S2. Subgroup analysis of PFS based on cytogenetic risk [15, 16, 25, 26, 28].

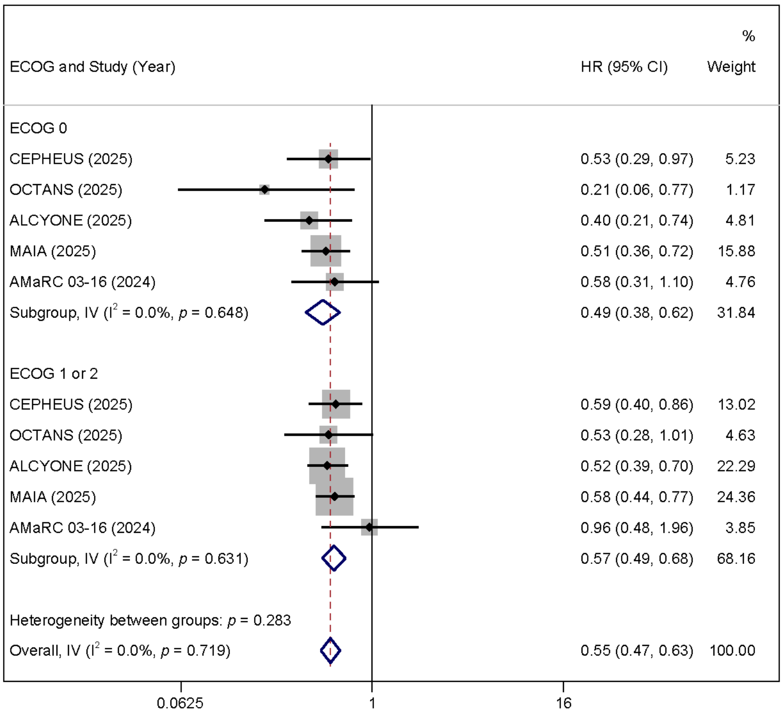

Figure S3. Subgroup analysis of PFS based on cytogenetic ECOG performance status [15, 16, 25, 26, 28].

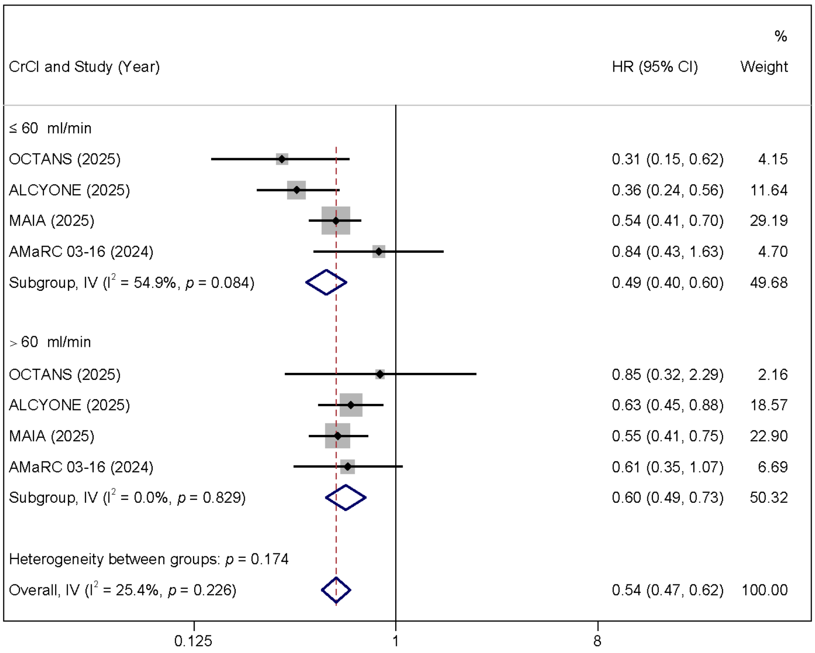

Figure S4. Subgroup analysis of PFS based on cytogenetic creatinine clearance [15, 16, 24, 26].

Table S1. PRISMA 2020 Checklist.

Section and Topic	Item #	Checklist item	Location where item is reported

Section and Topic	Item #	Checklist item	Location where item is reported
**TITLE**			
Title	1	Identify the report as a systematic review.	Page1, line1-3
**ABSTRACT**			
Abstract	2	See the PRISMA 2020 for Abstracts checklist.	Page1, line10-34
**INTRODUCTION**			
Rationale	3	Describe the rationale for the review in the context of existing knowledge.	Page2, line59-75
Objectives	4	Provide an explicit statement of the objective(s) or question(s) the review addresses.	Page2, line76-81
**METHODS**			
Eligibility criteria	5	Specify the inclusion and exclusion criteria for the review and how studies were grouped for the syntheses.	Page3, line91-96
Information sources	6	Specify all databases, registers, websites, organisations, reference lists and other sources searched or consulted to identify studies. Specify the date when each source was last searched or consulted.	Page2, line85-90
Search strategy	7	Present the full search strategies for all databases, registers and websites, including any filters and limits used.	Page2, line88-90
Selection process	8	Specify the methods used to decide whether a study met the inclusion criteria of the review, including how many reviewers screened each record and each report retrieved, whether they worked independently, and if applicable, details of automation tools used in the process.	Page3, line99-100
Data collection process	9	Specify the methods used to collect data from reports, including how many reviewers collected data from each report, whether they worked independently, any processes for obtaining or confirming data from study investigators, and if applicable, details of automation tools used in the process.	Page3, line99-103
Data items	10a	List and define all outcomes for which data were sought. Specify whether all results that were compatible with each outcome domain in each study were sought (e.g. for all measures, time points, analyses), and if not, the methods used to decide which results to collect.	Abstract, Methods and Page3, line99-103
	10b	List and define all other variables for which data were sought (e.g. participant and intervention characteristics, funding sources). Describe any assumptions made about any missing or unclear information.	Page3, line100-103
Study risk of bias assessment	11	Specify the methods used to assess risk of bias in the included studies, including details of the tool(s) used, how many reviewers assessed each study and whether they worked independently, and if applicable, details of automation tools used in the process.	Page3, line102-103
Effect measures	12	Specify for each outcome the effect measure(s) (e.g. risk ratio, mean difference) used in the synthesis or presentation of results.	Page3, line106-109
Synthesis methods	13a	Describe the processes used to decide which studies were eligible for each synthesis (e.g. tabulating the study intervention characteristics and comparing against the planned groups for each synthesis (item #5)).	Results, 3.2 (Explanati on for excluding GEM-2017 FIT from PFS analysis)
	13b	Describe any methods required to prepare the data for presentation or synthesis, such as handling of missing summary statistics, or data conversions.	Page3, line106-107
	13c	Describe any methods used to tabulate or visually display results of individual studies and syntheses.	Page3, line115 & Figures 2, 3, 4, 5; Table 1, 2
	13d	Describe any methods used to synthesize results and provide a rationale for the choice(s). If	Page3,

Section and Topic	Item #	Checklist item	Location where item is reported
		meta-analysis was performed, describe the model(s), method(s) to identify the presence and extent of statistical heterogeneity, and software package(s) used.	line109-114
	13e	Describe any methods used to explore possible causes of heterogeneity among study results (e.g. subgroup analysis, meta-regression).	Page3, line114-115
	13f	Describe any sensitivity analyses conducted to assess robustness of the synthesized results.	Page3, line115-118
Reporting bias assessment	14	Describe any methods used to assess risk of bias due to missing results in a synthesis (arising from reporting biases).	Page3, line118-12
Certainty assessment	15	Describe any methods used to assess certainty (or confidence) in the body of evidence for an outcome.	Not reported
**RESULTS**			
Study selection	16a	Describe the results of the search and selection process, from the number of records identified in the search to the number of studies included in the review, ideally using a flow diagram.	Results, 3.1 & Figure 1
	16b	Cite studies that might appear to meet the inclusion criteria, but which were excluded, and explain why they were excluded.	Figure 1
Study characteristics	17	Cite each included study and present its characteristics.	Results, 3.1 & Table 1
Risk of bias in studies	18	Present assessments of risk of bias for each included study.	Results, 3.5 & Figure 5
Results of individual studies	19	For all outcomes, present, for each study: (a) summary statistics for each group (where appropriate) and (b) an effect estimate and its precision (e.g. confidence/credible interval), ideally using structured tables or plots.	Figures 2, 3, 4
Results of syntheses	20a	For each synthesis, briefly summarise the characteristics and risk of bias among contributing studies.	Results, 3.2, 3.3, 3.5
	20b	Present results of all statistical syntheses conducted. If meta-analysis was done, present for each the summary estimate and its precision (e.g. confidence/credible interval) and measures of statistical heterogeneity. If comparing groups, describe the direction of the effect.	Results, 3.2, 3.3 & Figures 2, 3, 4; Table 2
	20c	Present results of all investigations of possible causes of heterogeneity among study results.	Results, 3.4
	20d	Present results of all sensitivity analyses conducted to assess the robustness of the synthesized results.	Results, 3.4 & Supplementary Table 1
Reporting biases	21	Present assessments of risk of bias due to missing results (arising from reporting biases) for each synthesis assessed.	As mentioned in Methods, publication bias was not formally assessed due to the small number of studies
Certainty of evidence	22	Present assessments of certainty (or confidence) in the body of evidence for each outcome assessed.	Not reported
**DISCUSSION**			

Section and Topic	Item #	Checklist item	Location where item is reported
Discussion	23a	Provide a general interpretation of the results in the context of other evidence.	Discussion, 4 (Paragraphs 1, 2)
	23b	Discuss any limitations of the evidence included in the review.	Discussion, 4 (Paragraph 6)
	23c	Discuss any limitations of the review processes used.	Discussion, 4 (Paragraph 6)
	23d	Discuss implications of the results for practice, policy, and future research.	Discussion, 4 (Paragraphs 5, 7)
**OTHER INFORMATION**			
Registration and protocol	24a	Provide registration information for the review, including register name and registration number, or state that the review was not registered.	Materials and Methods, 2.1
	24b	Indicate where the review protocol can be accessed, or state that a protocol was not prepared.	A protocol was not prepared
	24c	Describe and explain any amendments to information provided at registration or in the protocol.	The protocol is described in the Methods. Registration does not apply
Support	25	Describe sources of financial or non-financial support for the review, and the role of the funders or sponsors in the review.	Funding section
Competing interests	26	Declare any competing interests of review authors.	Conflicts of Interest section
Availability of data, code and other materials	27	Report which of the following are publicly available and where they can be found: template data collection forms; data extracted from included studies; data used for all analyses; analytic code; any other materials used in the review.	Data Availability Statement

Table S2. Leave-one-out sensitivity analysis of outcomes.

Outcomes	Excluded study	Pooled effect sizes			Heterogeneity	
		HR[a]/RR[b]	95%CI	*p*-value	I^2	*p*-value of Q test
PFS	AMaRC 03-16 [26]	0.534	0.473-0.604	< 0.001	34.6%	0.205
	MAIA [14,15]	0.541	0.467-0.625	< 0.001	46.3%	0.134
	ALCYONE [16,17]	0.535	0.462-0.620	< 0.001	45.1%	0.141
	OCTANS [24,25]	0.566	0.501-0.640	< 0.001	0.0%	0.870
	CEPHEUS [28]	0.540	0.476-0.613	< 0.001	45.6%	0.138
OS	AMaRC 03-16 [26]	0.673	0.587-0.771	< 0.001	0.0%	0.623

	MAIA [14,15]	0.711	0.602-0.840	< 0.001	45.3%	0.139
	ALCYONE [16,17]	0.725	0.609-0.864	< 0.001	41.5%	0.162
	OCTANS [24,25]	0.699	0.609-0.802	< 0.001	45.2%	0.140
	CEPHEUS [28]	0.673	0.584-0.776	< 0.001	33.2%	0.213
MRD negative rate	AMaRC 03-16 [26]	2.274	1.422-3.638	0.001	91.9%	< 0.001
	MAIA [14,15]	2.202	1.348-3.598	0.002	89.4%	< 0.001
	GEM-2017FIT [27]	2.775	1.729-4.454	< 0.001	83.8%	< 0.001
	ALCYONE [16,17]	2.016	1.314-3.093	0.001	87.2%	< 0.001
	OCTANS [24,25]	2.142	1.336-3.436	0.002	91.9%	< 0.001
	CEPHEUS [28]	2.629	1.378-5.016	0.003	91.9%	< 0.001
Sustained MRD negative rate	MAIA [14,15]	3.999	1.443-11.079	0.008	83.0%	0.003
	ALCYONE [16,17]	3.781	1.489-9.602	0.005	84.6%	0.001
	OCTANS [24,25]	3.351	1.624-6.916	0.001	85.3%	0.001
	CEPHEUS [28]	5.042	3.348-7.593	< 0.001	0.0%	0.417
Grade 3/4 AEs	AMaRC 03-16 [26]	1.066	1.022-1.111	0.003	0.0%	0.636
	MAIA [14,15]	1.062	0.988-1.142	0.104	0.0%	0.653
	ALCYONE [16,17]	1.089	1.041-1.139	< 0.001	0.0%	0.827
	OCTANS [24,25]	1.068	1.019-1.118	0.006	0.0%	0.613
Fatal AEs	AMaRC 03-16 [26]	1.427	1.095-1.861	0.009	30.8%	0.216
	MAIA [14,15]	1.695	1.215-2.365	0.002	0.0%	0.450
	GEM-2017FIT	1.339	1.020-1.757	0.035	0.0%	0.643
	ALCYONE [16,17]	1.378	1.008-1.884	0.044	28.2%	0.234
	OCTANS [24,25]	1.470	1.121-1.926	0.005	25.7%	0.250
	CEPHEUS [28]	1.399	1.026-1.909	0.034	30.3%	0.220
SAEs	AMaRC 03-16 [26]	1.144	1.063-1.231	< 0.001	7.2%	0.357
	MAIA [14,15]	1.184	1.048-1.338	0.007	9.2%	0.347
	ALCYONE [16,17]	1.120	1.042-1.203	0.002	0.0%	0.685
	OCTANS [24,25]	1.131	1.050-1.219	0.001	0.0%	0.549
	CEPHEUS [28]	1.172	1.073-1.279	< 0.001	0.0%	0.405
Grade 3/4 Lymphopenia	MAIA [14,15]	1.188	0.898-1.571	0.227	0.0%	0.887
	ALCYONE [16,17]	1.356	1.046-1.758	0.021	0.0%	0.813
	OCTANS [24,25]	1.280	0.996-1.645	0.054	0.0%	0.606
	CEPHEUS [28]	1.304	1.023-1.663	0.032	0.0%	0.633
Grade 3/4 Neutropenia	MAIA [14,15]	1.198	0.888-1.616	0.237	80.4%	0.002
	GEM-2017FIT [27]	1.175	0.906-1.525	0.225	82.3%	0.001
	ALCYONE [16,17]	1.322	0.996-1.755	0.054	79.2%	0.002
	OCTANS [24,25]	1.373	1.105-1.706	0.004	73.2%	0.011
	CEPHEUS [28]	1.202	0.901-1.604	0.211	84.0%	< 0.001
Grade 3/4 Thrombocytopenia	MAIA [14,15]	0.952	0.826-1.098	0.503	0.0%	0.930
	GEM-2017FIT [27]	0.947	0.822-1.090	0.448	0.0%	0.939
	ALCYONE [16,17]	0.981	0.812-1.186	0.844	0.0%	0.983
	OCTANS [24,25]	0.937	0.806-1.091	0.403	0.0%	0.988
	CEPHEUS [28]	0.950	0.816-1.107	0.513	0.0%	0.930
Grade 3/4 Anemia	MAIA [14,15]	1.032	0.826-1.290	0.781	29.2%	0.237
	GEM-2017FIT [27]	0.889	0.740-1.067	0.207	0.0%	0.698
	ALCYONE [16,17]	0.946	0.759-1.177	0.617	49.8%	0.113
	OCTANS [24,25]	0.941	0.777-1.140	0.536	49.7%	0.113
	CEPHEUS [28]	0.914	0.756-1.105	0.352	44.4%	0.352
Grade 3/4 Infection	MAIA [14,15]	1.418	1.023-1.965	0.036	71.7%	0.014
	GEM-2017FIT [27]	1.482	1.161-1.892	0.002	68.5%	0.023
	ALCYONE [16,17]	1.312	1.130-1.523	< 0.001	7.6%	0.355
	OCTANS [24,25]	1.411	1.075-1.852	0.013	71.5%	0.014
	CEPHEUS [28]	1.545	1.230-1.941	< 0.001	49.6%	0.114

Grade 3/4 Pneumonia	MAIA [14,15]	1.729	0.852-3.507	1.129	83.0%	0.003
	ALCYONE [16,17]	1.444	1.052-1.981	0.023	32.9%	0.225
	OCTANS [24,25]	1.946	1.058-3.580	0.032	80.3%	0.006
	CEPHEUS [28]	2.04	1.198-3.475	0.009	75.0%	0.018

[a] Hazard ratio (HR) was used as the measure of effect for time-to-event outcomes (PFS and OS)

[b] Risk ratio (RR) was used as the measure of effect for all dichotomous outcomes (MRD negativity rate, AEs, and other safety endpoints).
